# Prognostic and Clinical Significance of Cyclooxygenase-2 Overexpression in Endometrial Cancer: A Meta-Analysis

**DOI:** 10.3389/fonc.2020.01202

**Published:** 2020-08-06

**Authors:** Mingli Li, Mingxuan Li, Yangang Wei, Hua Xu

**Affiliations:** ^1^Department of Life Science and Engineering, Jining University, Jining, China; ^2^Nursing Department, Affiliated Hospital of Jining Medical University, Jining, China; ^3^Cisen Pharmaceutical Co., Ltd. Drug Discovery, Jining, China; ^4^Neurosurgery Department, Affiliated Hospital of Jining Medical University, Jining, China

**Keywords:** cyclooxygenase-2, endometrial cancer, prognosis, clinical significance, meta-analysis

## Abstract

**Objective:** Cyclooxygenase-2 (COX-2) is a critical enzyme associated with inflammation and tumorigenesis. Although several studies have compared the expression of COX-2 in endometrial cancer tissues and normal tissues, the results have been inconsistent thus far. This study aims to conduct a meta-analysis to elucidate the role of COX-2 in the determination of the risk, prognosis, and clinical features of endometrial cancer.

**Methods:** We retrieved the suitable studies on the association between COX-2 expression and endometrial cancer from PubMed, EMBASE, and Web of Science databases that were published between 1999 and September 31st, 2019. The hazard ratio (HR) and 95% confidence intervals (CIs) were retrieved to assess the relationship between COX-2 expression and the prognosis of endometrial cancer. The odds ratio (OR) and 95% CIs were calculated to evaluate the correlation between COX-2 expression and the risk and clinical features of endometrial cancer.

**Results:** To investigate the association between COX-2 expression and the susceptibility, clinical features, and prognosis of endometrial cancer, we performed a meta-analysis on data from selected studies that collectively involved 273 normal individuals and 1,376 patients with endometrial cancer. Overall, the pooled analysis indicated that COX-2 expression was significantly associated with susceptibility (Caucasians, OR = 3.94, 95% CI = 2.17–7.17, *P* < 0.05; Asians, OR = 20.51, 95% CI = 8.54–49.26, *P* < 0.05), cancer stage (OR = 3.01, 95% CI = 1.95–4.67, *P* < 0.05), myometrial invasion (OR = 1.59, 95% CI = 1.17–2.15, *P* < 0.05), lymph node metastasis (OR = 1.63, 95% CI = 1.18–2.26, *P* < 0.05), and prognosis (OR = 2.91, 95% CI = 1.17–4.66, *P* < 0.05) in endometrial cancer.

**Conclusions:** Our findings suggested that COX-2 overexpression is significantly associated with poor prognosis and advanced clinical features in endometrial cancer. Therefore, COX-2 may function as an effective prognostic biomarker and a potential therapeutic target for endometrial cancer.

## Introduction

Endometrial cancer is the most common gynecological cancer in developed countries, and the estimated mortality in the United States in 2017 was 10,920 (1, 2). Irregular or post-menopausal bleeding can be an early symptom of endometrial cancer that should prompt women to seek medical review, facilitating the early detection and timely treatment of endometrial cancer. However, many women still do present at an advanced stage.

However, the mortality rate of endometrial cancer has increased rapidly in recent years (3, 4). Several factors, including diagnosis at an older age, higher diagnostic rate of advanced-stage cancer, high-risk histology, and the rapid progression of endometrial cancer, may attribute to the high mortality rate of endometrial cancer. Patients with advanced endometrial cancer frequently have poor outcome, largely due to a paucity of effective treatment options.

According to an epidemiological survey, unopposed estrogen therapy, early menarche, late menopause, tamoxifen therapy, nulliparity, family history of endometrial cancer, age above 50 years, hypertension, obesity, thyroid disease, infertility, and polycystic ovary syndrome increased the risk of endometrial cancer (5, 6). Among the stated factors, obesity is of particular importance because nearly 70% of early stage endometrial cancer patients are obese (7).

Although these factors affect the development of endometrial cancer, no effective markers have been discovered to evaluate the risk of endometrial cancer in normal individuals. Therefore, more effective markers should be identified and employed. Recently published studies have identified several biological molecules that play a critical role in endometrial cancer development. The Cancer Genome Atlas Project has characterized several specific mutations in endometrial cancer (8). For instance, mutations in *PTEN, CTNNB1, ARID1A, PIK3CA*, and *KRAS* are commonly detected in endometrial cancer patients, and these mutations might suppress gene expression (9). According to published reports, up to 90% of endometrial cancer tissues exhibit low *PTEN* expression. PTEN is an important element of the PI3K pathway, which affects the activity of the mammalian target of rapamycin (mTOR). mTOR activation promotes cell growth and inhibits apoptosis (10). Furthermore, ARID1A, CTNNB1, FGFR2, FER2, and p53 have been observed to be expressed at abnormal levels in most cases of endometrial cancer (3). These mutations could result in the loss or gain of gene function. Certain studies have also demonstrated that chemotherapy drugs exerted different therapeutic effects in a population with these gene mutations. These studies have identified several potential and useful biomarkers for the treatment and prognosis of endometrial cancer.

Cyclooxygenase-2 is a rate-limiting enzyme that catalyzes the biosynthesis of prostaglandins (PGs) from arachidonic acid. The findings of basic *in vivo* and *in vitro* studies have suggested the significant association between COX-2 expression and cancer development (11, 12). COX-2 is expressed constitutively in normal tissues, and a higher expression of COX-2 has been detected in cancer tissues (13). *COX2* is an early response gene and can be induced by oncogenes, tumor promoters, and carcinogens. Up to 85% of colorectal adenocarcinoma patients were found to have high COX-2 expression. COX-2 affected the production of prostaglandins and also promoted the expression of MMP-2, MMP-9, and VEGF in colorectal cancer patients (14). Moreover, COX-2 was found to be significantly upregulated in gastric cancer, skin cancer, prostate cancer, and breast cancer tissues compared to normal tissues or adjacent tissues (15). Immunohistochemical analysis also revealed that COX-2 expression was significantly associated with the risk and development of endometrial cancer. However, the prognostic significance of COX-2 expression and its status as an early or late event in endometrial carcinoma remains controversial. Therefore, this meta-analysis was performed to assess the definite effect of COX-2 in the development and prognosis of endometrial cancer.

## Methods

### Literature Search Strategy

The eligible studies published between 1999 and September 31st, 2019 were retrieved from PubMed, EMBASE, and Web of Science databases following the Preferred Reporting Items for Systematic Reviews and Meta-analyses (PRISMA) guidelines (16). The following search strategy was used to retrieve relevant literature: “((“Endometrial Neoplasms”[Mesh]) AND (“Cyclooxygenase 2”[Mesh])) OR ((“Survival”[Mesh]” OR “Prognosis”[Mesh]”) and “endometrial cancer AND COX-2).”

### Study Selection

The inclusion criteria were as follows:

1) studies that assessed the relationship between COX-2 expression and endometrial cancer;

2) studies involving endometrial cancer patients who were diagnosed according to the relevant diagnostic criteria;

3) studies that had adequate data [COX-2 expression levels in case and control groups, hazard ratios (HR), and 95% confidence intervals (CI)] to estimate the pooled odds ratio (OR) and HR.

The exclusion criteria were as follows:

1) reviews, case reports, editorials, conference records, and comments;

2) studies that were published in non-English languages.

Since this was a meta-analysis, no ethical approval and patient consent were required.

### Data Extraction and Quality Assessment

Two investigators independently scanned the full-text articles and extracted the available data. The first author name, number of patients, year of publication, country, cancer type, race, follow-up duration, clinicopathological features of endometrial cancer patients, HRs, and 95% CI were extracted from the eligible studies. Furthermore, each study was scored on a scale of 0 to 9 according to the Newcastle–Ottawa scale (NOS) to evaluate the methodological quality. A study with a score <6 was considered low-quality, while that with a score ≥ 6 was considered methodologically sound. If conflicting results were observed, the two authors would discuss and reach a consensus.

### Statistical Analysis

Stata 14.0 (StataCorp, College Station, TX, USA) was used to analyze the extracted data. We used the ORs and 95% CI to evaluate the association between COX-2 expression and the susceptibility and clinical features of endometrial cancer. Moreover, the HRs and 95% CI were applied to assess the effect of COX-2 expression on the prognosis of endometrial cancer. Cochran's Q test and Higgins *I*^2^ statistic were used to estimate the heterogeneity among studies (17, 18), and an *I*^2^ ≥ 50% and *P* ≤ 0.05 were considered as parameters for significant heterogeneity. The random-effects model was used for meta-analysis when the heterogeneity among studies was significant; otherwise, the fixed-effects model was used. Begg's test and Egger's linear regression test were conducted to evaluate the publication bias (19, 20). In addition, we conducted a subgroup analysis based on race, pathological types, and pT stage for the susceptibility and clinical features of endometrial cancer. Lastly, we conducted a sensitivity analysis to estimate the robustness of overall results. In all tests, differences with *P* < 0.05 were considered statistically significant.

## Results

### Search Process and Eligible Study Characteristics

**Figure 1:**
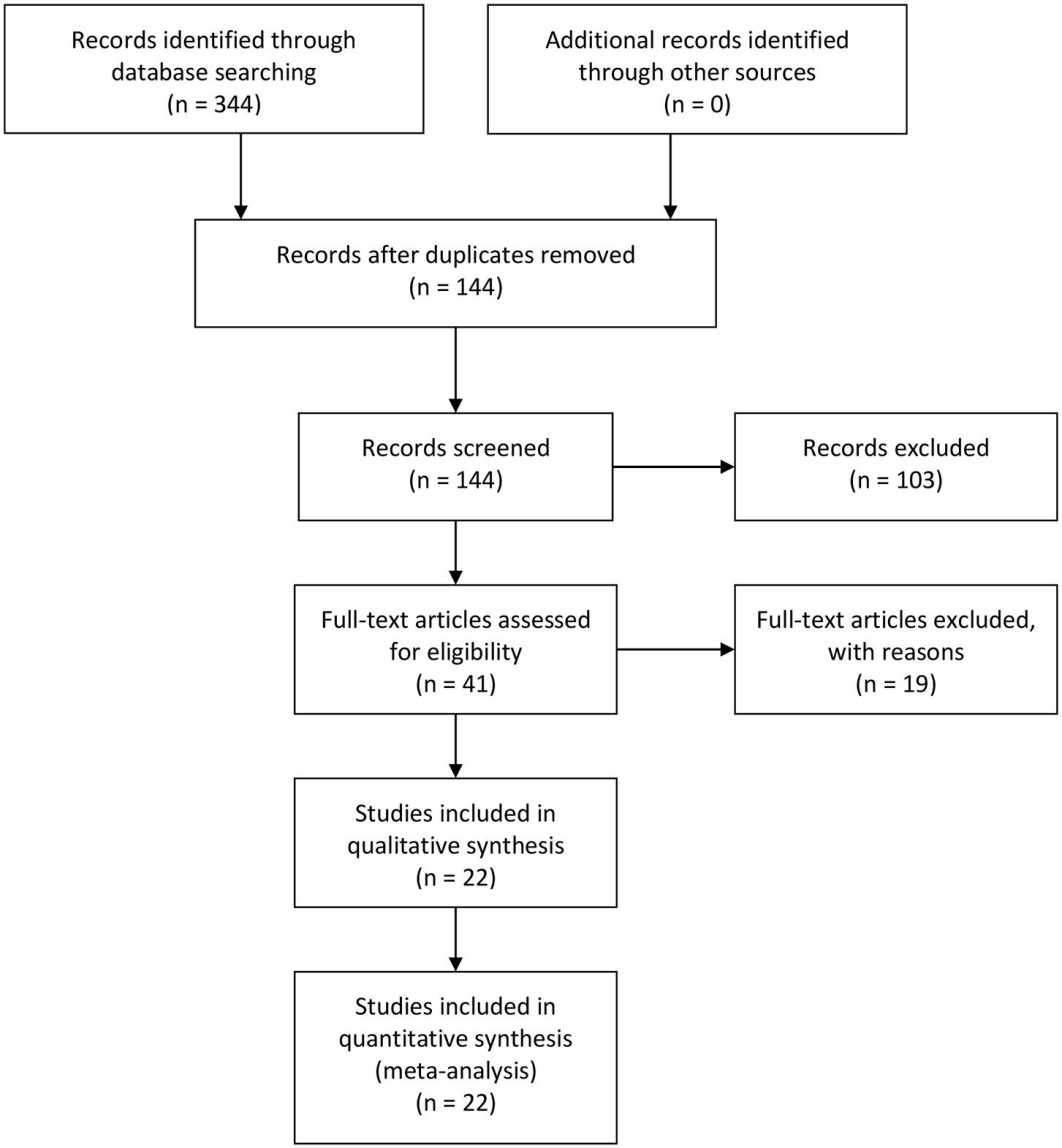
Flowchart of literature search.

Three hundred and forty-four relevant articles were retrieved from the initial search of PubMed, Web of Science, and EMBASE databases. After the title and abstract screening step, 200 publications were excluded, while 144 were retained. Next, the articles were read, and 175 publications were excluded for unusable data. Eventually, 22 studies involving 273 controls and 1,376 endometrial cancer patients were included in the meta-analysis (Figure 1) (21–42). The characteristics of all included studies were presented in Table 1. There were nine studies that evaluated the correlation between COX-2 expression and endometrial cancer susceptibility, of which five were conducted on Caucasians and four were conducted on Asians. Furthermore, there were 15 studies on the tumor grade of endometrial cancer, 10 on tumor stage, 14 on lymph node metastasis, and 9 on myometrial invasion (Supplementary Tables 1–4). Four studies on disease-free survival (DFS) were included in the analysis of endometrial cancer prognosis (Supplementary Table 5).

**Table 1:** Characteristics of included studies.

						**Normal tissue**		**Cancer tissue**			
**Author**	**Time**	**Country**	**Ethnicity**	**Method**	**Histology**	**COX-2 ^**−**^**	**COX-2 ^**+**^**	**COX-2 ^**−**^**	**COX-2 ^**+**^**	**NOS**	**Cut-off**
Landen et al. (21)	2003	USA	Caucasian	Immunofluorescent	Endometrial cancer	14	1	4	9	7	NR
Fowler et al. (22)	2005	USA	Caucasian	IHC	Endometrial cancer	73	32	138	198	6	10%
Hasegawa et al. (23)	2005	Japan	Asian	IHC	Endometrial cancer	9	1	23	26	6	5%
Kilic et al. (24)	2005	USA	Caucasian	IHC	Endometrial cancer	12	1	23	13	7	NR
Orejuela et al. (25)	2005	USA	Caucasian	IHC	Endometrial cancer	8	2	10	4	7	10%
Toyoki et al. (26)	2005	Japan	Asian	IHC	Endometrial cancer	20	0	48	2	6	NR
Chen and Liao (27)	2009	China	Asian	IHC	Endometrial cancer	25	5	8	43	7	0%
Jarzabek et al. (28)	2013	Poland	Caucasian	IHC	Endometrial cancer	4	6	6	45	6	10%
Ma et al. (29)	2015	China	Asian	IHC	Endometrial cancer	58	2	27	33	6	5%

*IHC, Immunohistochemistry; NOS, Newcastle-Ottawa Scale*.

### Associations of COX-2 Expression With Risk, Clinical Features, and Prognosis of Endometrial Cancer

**Figure 2:**
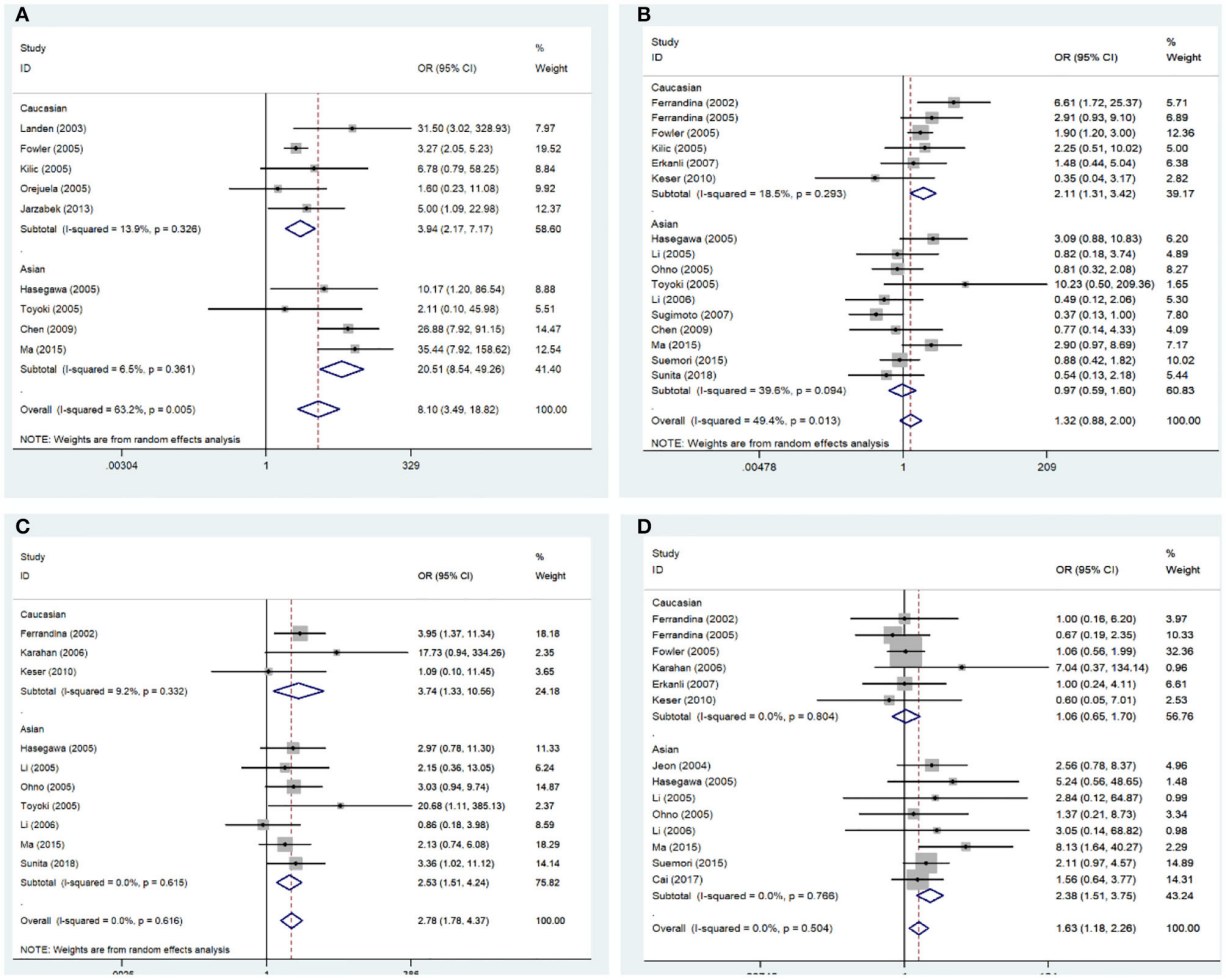
Forest plot of the association between COX-2 expression and the clinical features of endometrial cancer. **(A)** Endometrial cancer risk; **(B)** grade; **(C)** stage; **(D)** lymph node metastasis. OR, odds ratio; CI, confidence interval.

The correlations between COX-2 expression and susceptibility, clinical features, and prognosis of endometrial cancer was estimated; the pooled OR, HR, and 95% CI are presented in Table 2. COX-2 expression was significantly associated with DFS in endometrial cancer (HRs = 2.91; 95% CI = 1.17–4.66, *P* < 0.05). The association between COX-2 expression and endometrial cancer risk (HRs = 8.10; 95% CI = 3.49–18.82, *P* < 0.05) was assessed in nine studies. Owing to the significant heterogeneity, a subgroup analysis was conducted based on race, which revealed a significant association both in Caucasians and Asians (Caucasians, ORs = 3.94, 95% CI = 2.17–7.17, *P* < 0.05; Asians, ORs = 20.51, 95% CI = 8.54–49.26, *P* < 0.05). In addition, a subgroup analysis based on the pathological types revealed that COX-2 expression was associated with tumor stage (I vs. I-IV, ORs = 2.22, 95% CI = 1.22–4.02, *P* < 0.05), tumor grade in Caucasians (Caucasians, G1 vs. G2-G3, ORs = 2.11, 95% CI = 1.31–3.42, *P* < 0.05), and lymph node metastasis in Asians (Asians, negative vs. positive, ORs = 2.38, 95% CI = 1.51– 3.75, *P* < 0.05). However, the stratified analysis revealed that COX-2 expression was not related to the outer half myometrial invasion (ORs = 1.22, 95% CI = 0.62–2.38, *P* > 0.05) (Figure 2).

**Table 2:** Meta-analysis results for COX-2 expression in endometrial cancer.

				**Heterogeneity**		**Begg's test**		**Egger's test**	
**Characteristics**	**Studies**	**Pooled OR (95% CI)**	***P***	**I2 (%)**	***P***	**Z**	***P***	**T**	***P***
Risk	9	8.10 (3.49, 18.82)	<0.05	63.2	0.005	−0.21	0.835	1.42	0.2
Caucasian	5	3.94 (2.17, 7.17)	<0.05	13.9	0.326	1.96	0.05	1.08	0.36
Asian	4	20.51 (8.54, 49.26)	<0.05	6.5	0.3361	−1.36	0.174	−3.42	0.076
Tumor grade	15	1.32 (0.88, 2.00)	<0.05	49.4	0.013	−0.36	0.719	−0.34	0.737
Caucasian	6	2.11 (1.31, 3.42)	<0.05	18.5	0.293	−0.19	0.851	−0.01	0.996
Asian	10	0.97 (0.59, 2.00)	>0.05	39.6	0.094	0.27	0.788	0.77	0.465
Tumor stage	10	3.01 (1.95, 4.67)	<0.05	0	0.616	0.27	0.788	0.76	0.47
Caucasian	3	3.74 (1.33, 10.56)	<0.05	9.2	0.332	0.52	0.602	0.09	0.945
Asian	7	1.94 (1.00, 3.76)	<0.05	0	0.615	0.99	0.322	−1.9	0.106
Lymph node metastasis	14	1.63 (1.18, 2.26)	<0.05	0	0.766	0.93	0.352	1.19	0.258
Caucasian	6	1.06 (0.65, 1.70)	>0.05	0	0.804	0.56	0.573	0.43	0.686
Asian	8	2.38 (1.51, 3.75)	<0.05	0	0.766	0.49	0.621	1.24	0.263
Myometrial invasion	9	1.59 (1.17, 2.15)	<0.05	0	0.662	1.04	0.297	0.92	0.386
Caucasian	4	1.74 (1.16, 2.61)	<0.05	0	0.642	1.36	0.174	0.83	0.493
Asian	5	1.41 (0.89, 2.24)	>0.05	0	0.441	0.49	0.624	0.98	0.399
		Pooled HR (95% CI)							
Survival	4	2.91 (1.17, 4.66)	<0.05	0	0.858	0	1	2.21	0.157

*OR, odds ratio; HR, hazards ratio*.

### Publication Bias and Sensitivity Analysis

**Figure 3:**
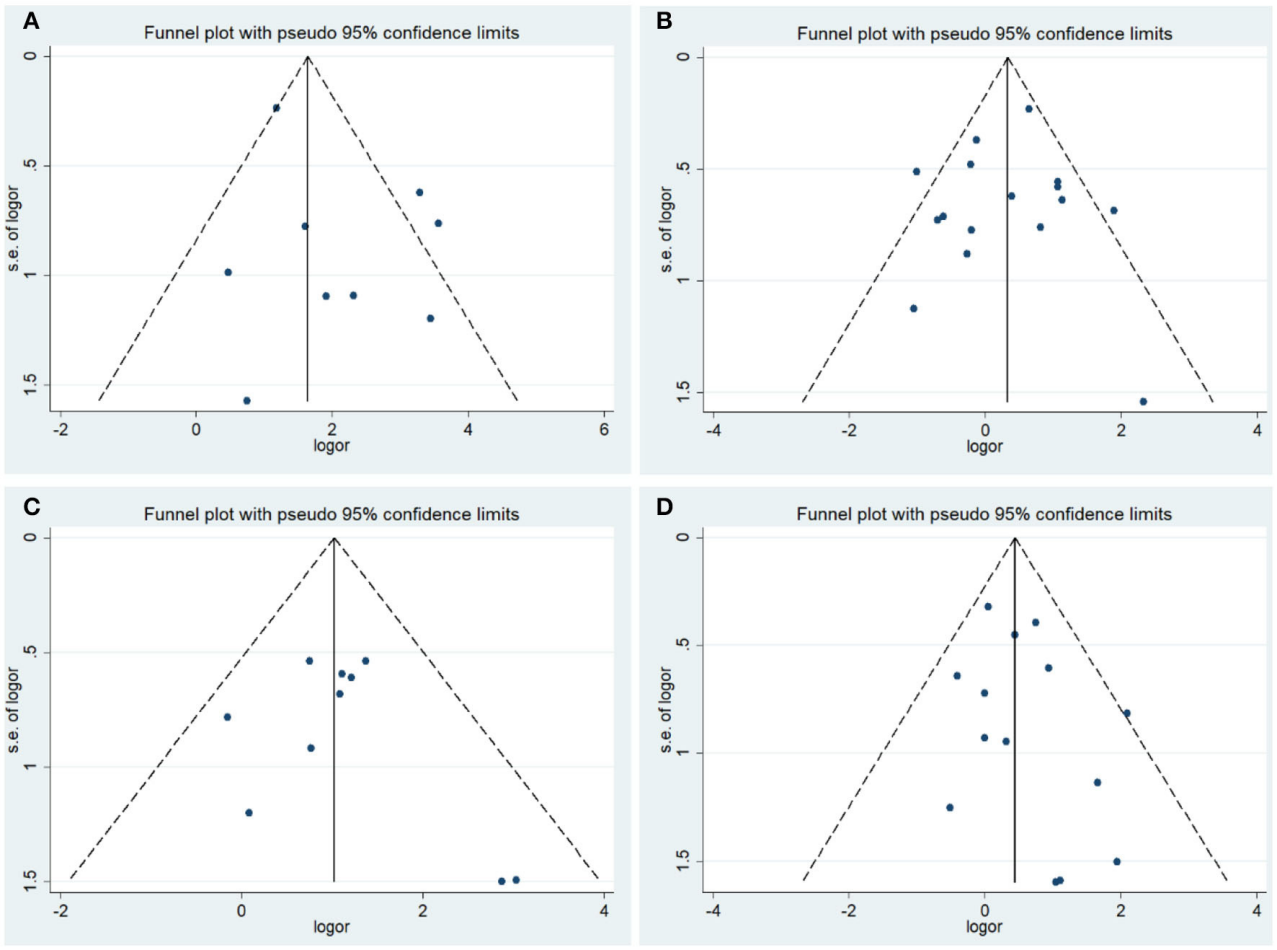
Funnel plot of the association between COX-2 expression and clinical features of endometrial cancer. **(A)** Endometrial cancer risk; **(B)** grade; **(C)** stage; **(D)** lymph node metastasis.

**Figure 4:**
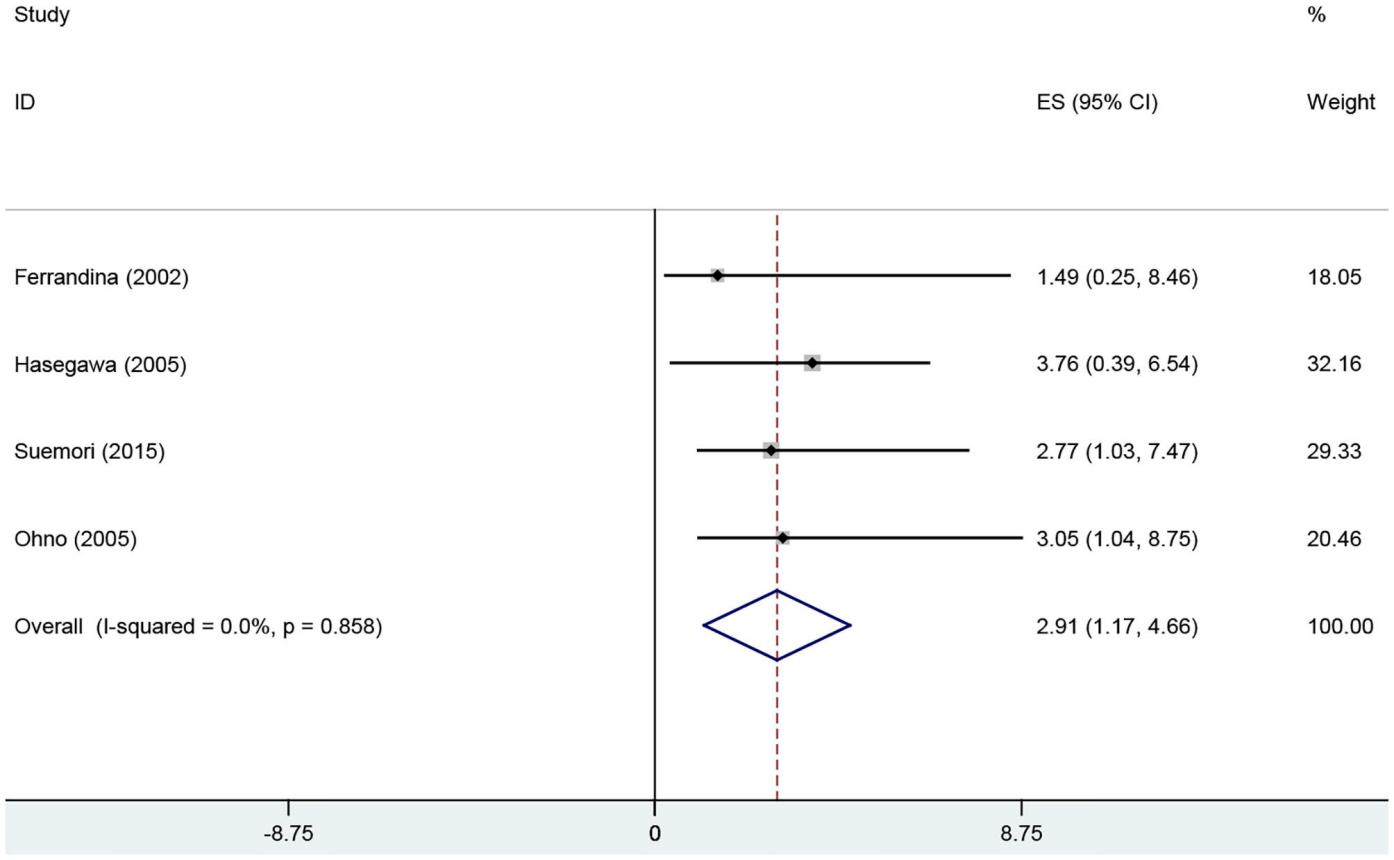
Forest plot of the association between COX-2 expression and DFS of endometrial cancer patients. DFS, disease-free survival.

The results of Begg's test and Egger's test did not provide evidence of publication bias among studies, except in those on tumor stage analysis, in which the Egger's test *P* < 0.05. To achieve more accurate results, a subgroup analysis based on race was conducted. Moreover, the results of the sensitivity analysis indicated that the overall ORs and HRs were stable (Figures 3, 4).

## Discussion

Several studies have been conducted over the past few decades to identify novel biomarkers for the early diagnosis and treatment of cancers. A wide variety of molecules, including proteins, metabolites, DNA, RNA, or cellular events, such as epigenetic alternations, could be used as biomarkers. These biomarkers might be used for risk assessment, screening, diagnosis, prognosis, treatment response prediction, and cancer monitoring in clinical practice (43). Cox-2 overexpression has been shown to induce tumorigenesis by promoting cell growth, angiogenesis, and metastasis of cancer cells in transgenic mice (44). Meanwhile, anti-inflammatory drugs could reduce COX-2 expression and gastrointestinal cancer mortality in a study (45). The prognostic significance of COX-2 overexpression has been evaluated in several cancers. In a study on prostate cancer, the inhibition of COX-2 prevented the growth of PC-3 tumors in nude mouse xenograft models (46). Additionally, the prostaglandin synthesis inhibitor also inhibited the production of MMP and reduced prostate tumor cell invasiveness (47). COX-2 also played a significant role in breast, lung, and ovarian cancers (15). In recent years, several studies have suggested that the COX-2 expression profile varied significantly between normal and endometrial cancer tissues. Additionally, COX-2 downregulation significantly inhibited the growth and invasiveness of the endometrial adenocarcinoma cell line HEC-1B (48, 49). Therefore, COX-2 might be a potential biomarker for the prognosis of endometrial cancer and a potential therapeutic target for its treatment. Here, we aimed to evaluate the significance of COX-2 in the development, progression, and prognosis of endometrial cancer.

To determine the stability of overall results and to remove heterogeneity from the study, we conducted a sensitivity analysis. There was significant heterogeneity in the overall analysis of endometrial cancer risk; to eliminate the same, a subgroup analysis based on race was performed and no heterogeneity was observed. Therefore, the results of the subgroup analysis were stable. In addition, a similar observation was made in the analysis of tumor grade of endometrial cancer, and the results of the subgroup analysis were robust. A significant heterogeneity was also observed in the other analysis. We excluded certain studies, such as those by Cai et al. (41) (tumor stage in Asians), Jeon et al. (31) (tumor grade in Asians), Keser et al. (38), Ferrandina et al. (30) (myometrial invasion in Caucasians), and Sugimoto et al. (37) (myometrial invasion in Asians), following which no significant heterogeneity was observed.

Based on results from 22 studies, we concluded that COX-2 overexpression predicted poor prognosis in endometrial cancer patients. The results of the Quantified Q test and *I*^2^ test indicated the absence of significant heterogeneity among studies in the survival analysis. Moreover, the results of the Begg' test and Egger's test suggested the absence of significant publication bias. Four studies were included to investigate the association between COX-2 expression and the prognosis of endometrial cancer, in which two studies yielded significant results (29, 34), and two studies yielded contradictory results (18, 25). Therefore, we could not confirm whether COX-2 expression was significantly associated with endometrial cancer prognosis based on data from previous studies. According to the meta-analysis, COX-2 overexpression might reduce the survival time of endometrial cancer patients. Relevant therapeutic interventions, which inhibited COX-2 activity, might be helpful in the treatment of endometrial cancer. However, to effectively avoid the restrictions imposed by a small sample size, more case-control studies should be conducted in future.

We also extracted clinical data from the included studies to explore the correlation between COX-2 expression and the progression of endometrial cancer. COX-2 expression was significantly associated with late-stage endometrial cancer, as well as with lymph node involvement in Asian populations. In previous studies, Ferrandina et al. reported that the frequency of COX-2 overexpression in late FIGO stage patients was higher than that in patients with <50% myometrial invasion (30, 32). Cao et al. reported that COX-2 overexpression was noted in moderately or poorly differentiated endometrioid cancer (50). In addition, the levels of COX-2 between pre- and post-menopausal patients were significantly different. These results suggested the potential value of COX-2 as a biomarker. In addition, the frequency of COX-2 overexpression in endometrial cancer patients was much higher than that in normal individuals. Collectively, no significant heterogeneity and publication bias were observed in the subgroup analyses, indicating the robustness of the results.

There were several limitations in this study. First, the cut-off value for COX-2 expression evaluation was inconsistent, and this might have introduced heterogeneity in the results. Second, we could not comprehensively analyze the role of COX-2 in endometrial cancer because the clinical information was considerably limited. Third, there was a limited number of patients with endometrial cancer in the eligible studies, which highlighted the importance of conducting more large-scale studies in future. Fourth, studies with positive results usually had higher chances of getting published than those with negative results, which might introduce a publication bias.

In general, our meta-analysis revealed the significant association between COX-2 overexpression and the poor prognosis and development of endometrial cancer. The results indicated that COX-2 overexpression might promote malignancy in endometrial cancer, and also suggested its significant association with increased susceptibility to endometrial cancer. Hence, determining the levels of COX-2 expression would help improve the diagnosis, treatment, and prognosis of endometrial cancer.

## Data Availability Statement

All datasets generated for this study are included in the article/Supplementary Material.

## Author Contributions

All authors listed have made a substantial, direct and intellectual contribution to the work, and approved it for publication.

## Conflict of Interest

YW was employed by Cisen Pharmaceutical Co., Ltd. Drug Discovery. The remaining authors declare that the research was conducted in the absence of any commercial or financial relationships that could be construed as a potential conflict of interest.
