# Supplementary material for: Prognostic and Clinical Significance of Cyclooxygenase-2 Overexpression in Endometrial Cancer: A Meta-Analysis
Source: Front Oncol. 2020 Aug 6;10:1202. doi: 10.3389/fonc.2020.01202 (PMC7438859; doi:10.3389/fonc.2020.01202)
Supplement: Supplementary file 1 [file Data_Sheet_1.docx]

**TABLE 1.** Main characteristics of eligible studies for the association COX-2 expression with grade of endometrial cancer.

|  |  |  |  |  |  | G1 | | G2-G3 | |  |
| --- | --- | --- | --- | --- | --- | --- | --- | --- | --- | --- |
| Author | Time | Country | Ethnicity | Method | Histology | COX-2 - | COX-2 + | COX-2 - | COX-2 + | Cut-off value |
| Ferrandina^31^ | 2002 | Italy | Caucasian | IHC | Endometrial carcinoma | 19 | 3 | 23 | 24 | 5 |
| Jeon^32^ | 2004 | Korea | Asian | IHC | Endometrial carcinoma | 74 | 14 | 14 | 13 | 10 |
| Ferrandina^33^ | 2005 | Italy | Caucasian | IHC | Endometrial carcinoma | 40 | 33 | 5 | 12 | 26% |
| Fowler^23^ | 2005 | USA | Caucasian | IHC | Endometrial carcinoma | 81 | 88 | 48 | 99 | 10 |
| Hasegawa^24^ | 2005 | Japan | Asian | IHC | Endometrial carcinoma | 18 | 14 | 5 | 12 | 5 |
| Kilic^25^ | 2005 | USA | Caucasian | IHC | Endometrial carcinoma | 18 | 8 | 5 | 5 | NR |
| Li^34^ | 2005 | China | Asian | IHC | Endometrial carcinoma | 5 | 11 | 5 | 9 | 0 |
| Ohno^35^ | 2005 | Japan | Asian | IHC | Endometrial carcinoma | 17 | 21 | 16 | 16 | 0 |
| Toyoki^27^ | 2005 | Japan | Asian | IHC | Endometrial carcinoma | 28 | 0 | 19 | 3 | NR |
| Li^36^ | 2006 | China | Asian | IHC | Endometrial carcinoma | 5 | 13 | 7 | 9 | 0 |
| Erkanli^37^ | 2007 | Turkey | Caucasian | IHC | Endometrial carcinoma | 7 | 8 | 13 | 22 | 0 |
| Sugimoto^38^ | 2007 | Japan | Asian | IHC | Endometrial carcinoma | 9 | 17 | 26 | 18 | NR |
| Chen^28^ | 2009 | China | Asian | IHC | Endometrial carcinoma | 2 | 13 | 6 | 30 | 0 |
| Keser^39^ | 2010 | Turkey | Caucasian | IHC | Endometrial carcinoma | 1 | 24 | 5 | 42 | 5 |
| Ma^30^ | 2015 | China | Asian | IHC | Endometrial carcinoma | 13 | 8 | 14 | 25 | 5 |
| Suemori^40^ | 2015 | Japan | Asian | IHC | Endometrial carcinoma | 32 | 22 | 43 | 26 | 5 |
| Sunita^41^ | 2018 | India | Asian | IHC | Endometrial carcinoma | 5 | 5 | 26 | 14 | 0 |
| Cai^42^ | 2017 | China | Asian | IHC | Endometrial carcinoma | 5 | 50 | 44 | 53 | 5 |

*IHC, Immunohistochemistry.*

**TABLE 2.** Main characteristics of eligible studies for the association COX-2 expression with stage of endometrial cancer.

|  |  |  |  |  |  | Ⅰ | | Ⅱ-Ⅳ | |
| --- | --- | --- | --- | --- | --- | --- | --- | --- | --- |
| Author | Time | Country | Ethnicity | Method | Histology | COX-2 - | COX-2 + | COX-2 - | COX-2 + |
| Ferrandina^31^ | 2002 | Italy | Caucasian | IHC | Endometrial carcinoma | 33 | 13 | 9 | 14 |
| Hasegawa^24^ | 2005 | Japan | Asian | IHC | Endometrial carcinoma | 19 | 16 | 4 | 10 |
| Li^34^ | 2005 | China | Asian | IHC | Endometrial carcinoma | 7 | 13 | 2 | 8 |
| Ohno^35^ | 2005 | Japan | Asian | IHC | Endometrial carcinoma | 28 | 24 | 5 | 13 |
| Toyoki^27^ | 2005 | Japan | Asian | IHC | Endometrial carcinoma | 25 | 0 | 18 | 7 |
| Karahan^43^ | 2006 | Turkey | Caucasian | IHC | Endometrial carcinoma | 7 | 16 | 0 | 19 |
| Li^36^ | 2006 | China | Asian | IHC | Endometrial carcinoma | 6 | 14 | 4 | 8 |
| Keser^39^ | 2010 | Turkey | Caucasian | IHC | Endometrial carcinoma | 5 | 23 | 1 | 5 |
| Ma^30^ | 2015 | China | Asian | IHC | Endometrial carcinoma | 18 | 16 | 9 | 17 |
| Cai^42^ | 2017 | China | Asian | IHC | Endometrial carcinoma | 22 | 59 | 27 | 44 |
| Sunita^41^ | 2018 | India | Asian | IHC | Endometrial carcinoma | 22 | 8 | 9 | 11 |

*IHC, Immunohistochemistry.*

**TABLE 3.** Main characteristics of eligible studies for the association COX-2 expression with lymph node metastasis of endometrial cancer.

|  |  |  |  |  |  | Negative | | Positive | |
| --- | --- | --- | --- | --- | --- | --- | --- | --- | --- |
| Author | Time | Country | Ethnicity | Method | Histology | COX-2 - | COX-2 + | COX-2 - | COX-2 + |
| Ferrandina^31^ | 2002 | Italy | Caucasian | IHC | Endometrial carcinoma | 10 | 10 | 3 | 3 |
| Jeon^32^ | 2004 | Korea | Asian | IHC | Endometrial carcinoma | 92 | 18 | 10 | 5 |
| Ferrandina^33^ | 2005 | Italy | Caucasian | IHC | Endometrial carcinoma | 28 | 30 | 7 | 5 |
| Fowler^23^ | 2005 | USA | Caucasian | IHC | Endometrial carcinoma | 101 | 146 | 19 | 29 |
| Hasegawa^24^ | 2005 | Japan | Asian | IHC | Endometrial carcinoma | 22 | 21 | 1 | 5 |
| Li^34^ | 2005 | China | Asian | IHC | Endometrial carcinoma | 10 | 18 | 0 | 2 |
| Ohno^35^ | 2005 | Japan | Asian | IHC | Endometrial carcinoma | 31 | 34 | 2 | 3 |
| Karahan^43^ | 2006 | Turkey | Caucasian | IHC | Endometrial carcinoma | 7 | 24 | 0 | 11 |
| Li^36^ | 2006 | China | Asian | IHC | Endometrial carcinoma | 12 | 20 | 0 | 2 |
| Erkanli^37^ | 2007 | Turkey | Caucasian | IHC | Endometrial carcinoma | 16 | 24 | 4 | 6 |
| Keser^39^ | 2010 | Turkey | Caucasian | IHC | Endometrial carcinoma | 5 | 25 | 1 | 3 |
| Ma^30^ | 2015 | China | Asian | IHC | Endometrial carcinoma | 25 | 20 | 2 | 13 |
| Suemori^40^ | 2015 | Japan | Asian | IHC | Endometrial carcinoma | 56 | 28 | 19 | 20 |
| Cai^42^ | 2017 | China | Asian | IHC | Endometrial carcinoma | 41 | 79 | 8 | 24 |

*IHC, Immunohistochemistry.*

**TABLE 4.** Main characteristics of eligible studies for the association COX-2 expression with myometrial invasion of endometrial cancer.

|  |  |  |  |  |  | No | | Yes | |
| --- | --- | --- | --- | --- | --- | --- | --- | --- | --- |
| Author | Time | Country | Ethnicity | Method | Histology | COX-2 - | COX-2 + | COX-2 - | COX-2 + |
| Ferrandina^31^ | 2002 | Italy | Caucasian | IHC | Endometrial carcinoma | 29 | 3 | 7 | 14 |
| Jeon^32^ | 2004 | Korea | Asian | IHC | Endometrial carcinoma | 97 | 18 | 28 | 9 |
| Ferrandina^33^ | 2005 | Italy | Caucasian | IHC | Endometrial carcinoma | 24 | 22 | 21 | 23 |
| Fowler^23^ | 2005 | USA | Caucasian | IHC | Endometrial carcinoma | 99 | 126 | 27 | 61 |
| Hasegawa^24^ | 2005 | Japan | Asian | IHC | Endometrial carcinoma | 18 | 13 | 5 | 13 |
| Li^34^ | 2005 | China | Asian | IHC | Endometrial carcinoma | 7 | 12 | 3 | 8 |
| Li^36^ | 2006 | China | Asian | IHC | Endometrial carcinoma | 8 | 14 | 4 | 8 |
| Karahan^43^ | 2006 | Turkey | Caucasian | IHC | Endometrial carcinoma | 5 | 15 | 2 | 20 |
| Erkanli^37^ | 2007 | Turkey | Caucasian | IHC | Endometrial carcinoma | 12 | 13 | 5 | 14 |
| Sugimoto^38^ | 2007 | Japan | Asian | IHC | Endometrial carcinoma | 5 | 15 | 30 | 20 |
| Keser^39^ | 2010 | Turkey | Caucasian | IHC | Endometrial carcinoma | 1 | 40 | 4 | 21 |
| Suemori^40^ | 2015 | Japan | Asian | IHC | Endometrial carcinoma | 36 | 24 | 39 | 24 |
| Cai^42^ | 2017 | China | Asian | IHC | Endometrial carcinoma | 18 | 77 | 29 | 17 |

*IHC, Immunohistochemistry.*

**TABLE 5.** Main characteristics of eligible studies for the association COX-2 expression with survival of endometrial cancer patients.

| Author | Time | Country | Ethnicity | Tumor stage | Detected sample | Num. | Follow-up median | Survival analysis | Source of HR | HR | 95%CI | P | cut-off | LL | UL |
| --- | --- | --- | --- | --- | --- | --- | --- | --- | --- | --- | --- | --- | --- | --- | --- |
| Ferrandina^26^ | 2002 | Italy | Caucasian | Ⅰ-Ⅳ | Endometrial carcinoma | 53 | 69 | DFS | Curve | 1.49 | 0.25-8.46 | 0.25 | 5% | 0.25 | 8.46 |
| Hasegawa^19^ | 2005 | Japan | Asian | Ⅰ-Ⅳ | Endometrial carcinoma | 49 | 72 | DFS | Curve | 3.76 | 0.39-6.54 | 0.35 | 5% | 0.39 | 6.54 |
| Ohno^30^ | 2005 | Japan | Asian | Ⅰ-Ⅳ | Endometrial carcinoma | 70 | 78 | DFS | Curve | 3.05 | 1.04-8.75 | 0.029 | 25% | 1.04 | 8.75 |
| Suemori^35^ | 2015 | Japan | Asian | Ⅰ-Ⅳ | Endometrial carcinoma | 123 | 72 | DFS | Curve | 2.77 | 1.03-7.47 | 0.038 | 5% | 1.03 | 7.47 |

*DFS, disease free survival; HR, hazards ratio; LL, lower limit; UL, upper limit.*
